# Deep learning fusion of satellite and social information to estimate human migratory flows

**DOI:** 10.1111/tgis.12953

**Published:** 2022-06-27

**Authors:** Daniel Runfola, Heather Baier, Laura Mills, Maeve Naughton‐Rockwell, Anthony Stefanidis

**Affiliations:** ^1^ Department of Applied Science William & Mary Williamsburg Virginia USA; ^2^ Geospatial Evaluation and Observation Laboratory William & Mary Williamsburg Virginia USA; ^3^ Initiative for Computational Societal and Security Research William & Mary Williamsburg Virginia USA; ^4^ Department of Computer Science William & Mary Williamsburg Virginia USA

## Abstract

Human migratory decisions are driven by a wide range of factors, including economic and environmental conditions, conflict, and evolving social dynamics. These factors are reflected in disparate data sources, including household surveys, satellite imagery, and even news and social media. Here, we present a deep learning‐based data fusion technique integrating satellite and census data to estimate migratory flows from Mexico to the United States. We leverage a three‐stage approach, in which we (1) construct a matrix‐based representation of socioeconomic information for each municipality in Mexico, (2) implement a convolutional neural network with both satellite imagery and the constructed socioeconomic matrix, and (3) use the output vectors of information to estimate migratory flows. We find that this approach outperforms alternatives by approximately 10% (*r* ^2^), suggesting multi‐modal data fusion provides a valuable pathway forward for modeling migratory processes.

**Abbreviations**
CNN: convolutional neural network
DL: deep learning

## 1 INTRODUCTION & LITERATURE REVIEW

Humans have been migrating for thousands of years, and over time the causes, patterns of manifestation, and effects of human migration have been evolving together with human society. Reflecting its complex nature, human migration has been studied through the lens of various disciplines including economics (Stark & Bloom, 1985), sociology (Castles, 2007), geography (King, 2011), political theory (Sager, 2016), and multidisciplinary integrative efforts (Nawrotzki et al., 2015).

Today, human migratory decisions are influenced by a large range of factors such as changing economic and environmental conditions (Black et al., 2011; Brettell & Hollifield, 2014; Clark, 1986; Hunter et al., 2015; Leyk et al., 2017), conflict (Abel et al., 2019; Burrows & Kinney, 2015), and evolving social dynamics (Dustmann et al., 2017; Mirilovic, 2010; Segal, 2019). Migratory decisions are commonly made at the household or individual level (Nawrotzki et al., 2015a), as a means to respond and adapt to the effects of the abovementioned factors on human livelihoods and well‐being (Brettell & Hollifield, 2014; Leyk et al., 2017). Occasionally, conditions lead to rapid increases in migrants arriving at a single destination within a small period of time (U.S. Customs and Border Protection, 2021a). Coupled with the complex legal frameworks that govern migratory inflows into most countries, such unexpected rapid increases of migrant populations can result in extremely long processing times, overwhelmed local authorities, increases in illicit border crossings, and—in extreme cases—mortality events (Androff & Tavassoli, 2012; Angelucci, 2012; U.S. Customs and Border Protection, 2021b; Délano Alonso & Nienass, 2016; Eschbach et al., 1999). Recently, this has been of particular concern at the border between the United States and Mexico, with considerable political and public attention being focused on the interplay between governmental policy and the well‐being of migrant populations (Abi‐Habib, 2021; Miroff, 2021).

Efforts to mitigate challenges associated with extreme variations of migratory flows commonly depend on: (1) improving our ability to forecast migratory flows to better allocate resources during anticipated periods of high migration activity; and (2) reducing migratory outflows by improving living conditions at migrant origin locales. In this context, scholars and practitioners have conducted research into the drivers of migration (Hanson & Spilimbergo, 1999; Hunter et al., 2015; Lindstrom & Lauster, 2001; Massey & Zenteno, 2000; McKenzie & Rapoport, 2010; Nawrotzki et al., 2015b; Riosmena, 2010; Runfola et al., 2016; Sue et al., 2019), including early exploratory efforts on the potential of satellite imagery to advance our understanding of migration dynamics, and the ability to predict patterns of migratory flows (Leyk et al., 2017; Nawrotzki et al., 2015a; Runfola et al., 2016; Runfola & Napier, 2016). One particular challenge in pursuing this research agenda has been the fact that migration‐relevant information is conveyed across many disparate sources, ranging from tabular datasets (i.e., household surveys) to satellite imagery and even news and social media.

Building on this literature, in this article, we specifically explore how survey and satellite data can be integrated within the convolutional stages of a deep learning model. This allows us to fully explore in an integrative manner suggestions offered individually in a variety of disciplines regarding causes of migration, to offer a robust and thorough study that contributes to this literature, and to advance our corresponding predictive capability. To accomplish this, we introduce a technique that transforms tabular (1D) census data into a meaningfully arranged matrix (2D) of information suitable for convolution. In the remainder of Section 1, we provide a review of the nascent literature exploring the use of satellite imagery and convolutional neural networks, as well as related data fusion strategies that have been pursued in other disciplines. In Section 2, we introduce our study area and datasets; in Section 3, we discuss our methodology and model workflow. Section 4 shows our results, and in Section 5 we provide a brief discussion of the potential for and challenges to this type of approach.

### 1.1 Convolutional neural networks and satellite imagery

For decades, satellite‐based methods have been used to quantify a wide range of land‐cover and land‐use characteristics based on observable image data (Fortier et al., 2011; Gao et al., 2011; Griffin et al., 2011; Jensen, 1981; Jensen, 1983; Polsky et al., 2012; Rogan et al., 2004, 2010; Runfola, 2012; Runfola et al., 2014). In this context, the last decade has seen a rapid emergence of interest specifically in convolutional neural networks for land‐cover and land‐use estimation, with a focus on scene classification algorithms (i.e., determining if a given collection of pixels represented a forest, water body, or residential building) (Hu et al., 2015; Li et al., 2018; Ma et al., 2019; Nogueira et al., 2017; Sumbul et al., 2019; Xia et al., 2017; Zhang et al., 2019). Progress in this emergent field has served to illustrate both the value of convolutional approaches and the many challenges to their success in the context of satellite imagery; a number of survey articles have recently attempted to capture the breadth of these (Cheng et al., 2020; Sumbul et al., 2019; Xia et al., 2017). A much smaller subset of the literature—while building on scene‐based classification—focuses on a more specific problem: estimating a continuous socioeconomic variable such as income on the basis of satellite imagery.

With the growth of convolutional neural network‐based approaches to satellite imagery analysis, studies are now beginning to emerge which seek to quantify explicit attributes about geographic locations—that is, the income of a household (Babenko et al., 2017; Jean et al., 2016; Perez et al., 2017; Tingzon et al., 2019), likelihood of a conflict event (Goodman et al., 2020), population density (Hu et al., 2019; Tiecke et al., 2017), school education outcomes (Runfola et al., 2021), and continuous grades of road quality (Brewer et al., 2021; Cadamuro et al., 2018). Many of these studies have been in response to the critical lack of data on human well‐being in data‐scarce environments (Burke et al., 2021), specifically seeking to improve our ability to capture relationships in impoverished areas (Jean et al., 2016). Among other contributions, this literature has established the value of transfer learning in overcoming the relatively small‐N of many socioeconomic datasets (Brewer et al., 2021; Goodman et al., 2020; Jean et al., 2016; Runfola et al., 2021).

These pathbreaking studies have illustrated the tremendous amount of information contained in satellite image data, reflective of long‐theorized relationships between the ways in which humans modify the landscape and underlying societal factors (Kugler et al., 2019; Runfola & Hughes, 2014). However, the information in satellite data is not unlimited: there are many social factors that cannot be adequately measured using imagery alone (Burke et al., 2021). One common approach to overcoming this limitation is through the integration of other data sources (i.e., tabular surveys) into deep learning models to improve overall predictive capability.

### 1.2 Data integration in convolutional neural networks

Convolutional neural networks (CNNs) have predominantly been applied to extract numeric vectors of data from imagery, where each vector contains information on the presence or absence of features of relevance for a particular algorithm task (i.e., identifying if a car is in an image) (Lecun et al., 2015). CNNs rely on a set of convolutional layers, in which each convolution involves shifting a moving window (the “filter”) across an image, and at each movement calculating the multiplicative sum of each filter weight and the underlying image data. After this process is completed, the filter weights themselves are then updated through an optimization routine, repeated iteratively until meaningful patterns are identified (Lecun et al., 2015). In most contexts, filter dimensions become iteratively smaller in deeper layers of the network, until an affine (or, fully connected) layer is utilized to produce a final score for a given input image. This final affine layer most commonly takes the form of a multi‐layer neural network in which all nodes are connected to all other nodes in the following layer (Lecun et al., 2015).

In the cases where ancillary data are used alongside imagery in a prediction (i.e., metadata providing the location of a cellphone), the ancillary information is generally integrated only in the final affine layer [in the context of satellite imagery, see e.g., Babenko et al. (2017), Burke et al. (2021), Cadamuro et al. (2018), Goodman et al. (2020), Hu et al. (2019), Jean et al. (2016), Perez et al. (2017), and Tingzon et al. (2019)]. In the broader literature, recent work has explored the integration of tabular data into the convolutional network itself, rather than only the final predictive layer(s). In 2019, Sharma et al. (2019) proposed a technique to arrange data about genes into meaningful clusters across a two‐dimensional surface, allowing the tabular information about those genes to be analyzed using convolutional approaches. By reprojecting 1D vectors into two‐dimensional space and employing convolutional models, they saw—on average—a 9% gain in accuracy as contrasted to current state‐of‐the‐art classification models. Separate, but related work focused on time‐series manipulation has established the value of transforming data (such as sensor inputs from robot‐mounted cameras) into 2D “fingerprints” for integration with other machine learning techniques (Hinders, 2020). Recent research has noted the value of such transformations for sparse datasets (Kanber, 2020), improving the performance of transfer learning approaches (Kovalerchuk & Agarwal, 2020), and increasing computational efficiency (Kanber, 2020; Kovalerchuk & Agarwal, 2020).

In this article, we build on this research to explore the value of integrating ancillary tabular data (census information) with satellite imagery data across all layers of a convolutional network. To do so, we implement a “social signature” approach to generate a dynamically generated 2D surface of socioeconomic variables that is suitable for convolution. This strategy builds on recent research from a range of disciplines indicating such a strategy can improve the networks ability to learn patterns (i.e., if certain variables interrelate with one another), but is as‐of‐yet untested in the context of satellite data (Kanber, 2020, Kovalerchuk & Agarwal, 2020; Sharma et al., 2019).

## 2 STUDY AREA AND DATA

### 2.1 Study area

**FIGURE 1:**
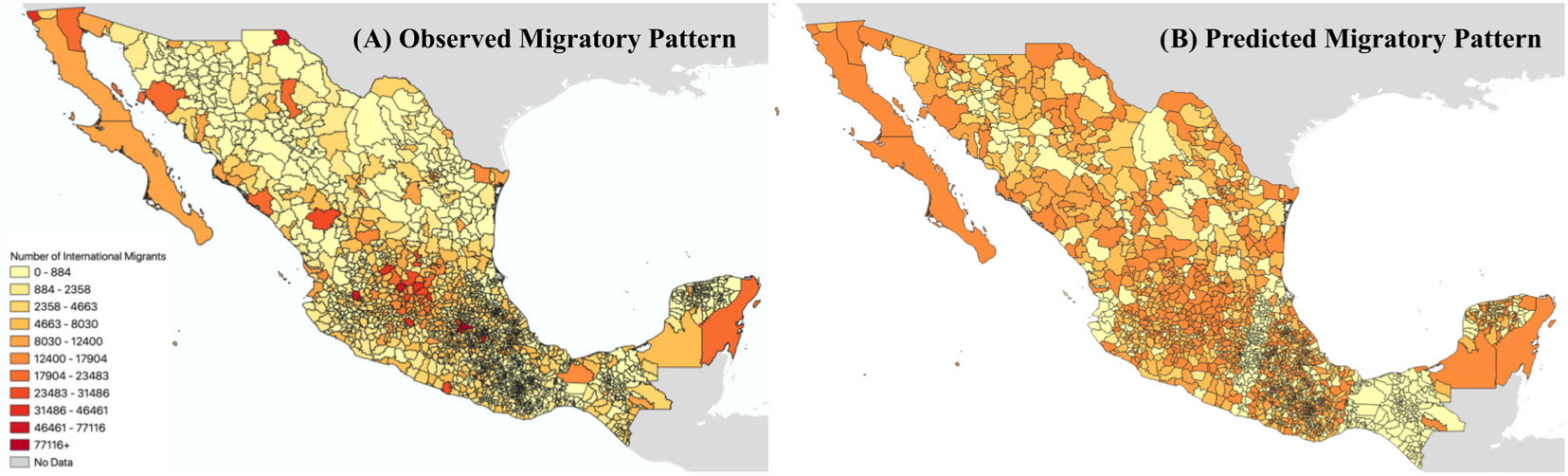
Map of the 2358 municipalities included in this analysis. Panel (a) shows the observed pattern of migration, with darker shades indicating a higher intensity of international migration. Panel (b) shows the predicted pattern of migration from the social signature model, with the same color scheme. Information is provided by IPUMS (Ruggles et al., 2003); map boundaries are provided by geoBoundaries (Runfola et al., 2020).

In this article, we seek to estimate the total number of international migrants leaving Mexico, with estimates focused on the specific municipality of departure (see Figure 1). In recent decades, Mexico has been the top origin country for immigrant populations moving to the United States; in 2018, 25% of all migrants to the United States originated in Mexico [with China representing the second most common origin, with 6% (Budiman, 2020)].

Our analysis is based on municipalities of Mexico, which represents the smallest geographic unit at which information regarding migratory flows are publicly available (Ruggles et al., 2003); we ultimately seek to estimate the number of migrants leaving a given municipality for an international destination. In 2010, the most recent decade for which census data are today available, there were 2358 municipalities in Mexico. Municipalities are considered second‐level administrative units (Runfola et al., 2020), and are led by an elected municipal council which provisions public services across each region.

### 2.2 Data

#### 2.2.1 Census information

Information on socioeconomic characteristics and migratory flows from each municipality in Mexico were collected from the 2010 Population and Housing Census conducted by the Instituto Nacional de Estadística, Geografía e Informática (INEGI), as distributed by IPUMS (Ruggles et al., 2003). The 2010 decennial census was conducted in Mexico between May and June of 2010, and was conducted with a 10% sample of the population (*N* = 11,938,402). A one‐stage stratified cluster sample was implemented by municipality, with specific enumeration areas selected by random sampling. Sample weights constructed based on the relative population sampled are provided by the government of Mexico, which allow for weighted aggregate statistics to be generated for each household and, in turn, municipality.

For our outcome variable, we rely on a survey question which indicates the number of people in a household who have—over the 5 years preceding the interview—left to go live in another country. Respondents were instructed to exclude events such as vacations, work assignments, visits to relatives, or other events that would not result in a change of residence (IPUMS International, 2021). This variable allows us to construct a per‐municipality estimate of international migrants between 2005 and 2010.^1^ We further integrate a large number (201) of ancillary variables from the 2010 Population and Housing Census into our analysis, which are used as the basis for the analysis we present in Section 3.2. These variables (aggregated to the municipality) are summarized in Table 1, and are standardized before use. A full list of all ancillary variables used in this analysis is provided in the Appendix 1.

**TABLE 1:** Representative selection of variables used in this analysis. Additional variables included (see the Appendix 1), for example, binary variables indicating the specific type of trash collection, or mechanism through which water entered a home

	Mean	Std	Min	Max
Weighted avg income	692,270.76	57,591.44	451,129.67	962,387.30
Total pop	48,030.95	191,678.79	90.00	5,210,265.00
% Rural	0.61	0.35	0.00	1.00
% Owned	0.86	0.09	0.46	1.00
% Yes electricity	0.95	0.06	0.30	1.00
% Electricity fuelcook	0.00	0.00	0.00	0.07
% Sewage system	0.44	0.32	0.00	0.99
% Yes cell	0.41	0.26	0.00	0.92
% Yes internet	0.07	0.09	0.00	0.66
% Yes autos	0.33	0.20	0.01	0.95
% Yes computer	0.13	0.11	0.00	0.70
Avg room num	3.40	0.54	1.95	5.89
Avg bedroom num	2.00	0.27	1.16	2.82
% Yes kitchen	0.86	0.11	0.28	1.00
% Flush toilet	0.38	0.27	0.00	0.98
% Non‐flush other toilet	0.53	0.27	0.01	1.00
% Married with children hhtype	0.49	0.07	0.22	0.74
% Married	0.42	0.03	0.31	0.54
Avg nfams	1.03	0.02	1.00	1.32
Avg nmothers	1.11	0.11	0.69	2.13
% Single parent hhtype	0.07	0.02	0.01	0.17
% Single	0.50	0.04	0.34	0.64
% Yes school	0.28	0.04	0.15	0.43
Avg years of school	5.16	1.13	1.85	9.73
% Unemployed	0.01	0.01	0.00	0.11
% Disabled	0.02	0.01	0.00	0.12
% Electricity fuelcook	0.00	0.00	0.00	0.07
% Other fuelcook	0.00	0.00	0.00	0.02
% Gas piped utility fuelcook	0.13	0.18	0.00	0.81
% Gas tanked bottled fuelcook	0.53	0.33	0.00	1.00
% Wood fuelcook	0.45	0.34	0.00	1.00
% Charcoal fuelcook	0.01	0.01	0.00	0.14

#### 2.2.2 Satellite information

The Landsat 5 Thematic Mapper (TM) Level‐1 data product (USGS, 2021) is leveraged in this study. This product provides level‐1 precision terrain (L1TP), inter‐calibrated data, and georegistration errors with a root mean square error of less than 12 m. For each municipality in Mexico, we estimate a cloud‐free monthly scene by compositing all images taken within a given calendar month by either: (a) selecting and taking the minimum of all cloud‐free pixels; or (b) masking pixels that have no cloud‐free imagery available for the selected time period (Google, 2021). Following this approach, we retrieve imagery for each municipality in Mexico, for the month of January in calendar year 2010 (selected to align with relevant growing seasons). Each municipalities' imagery is subdivided into tiles with 224 pixels on a side,^2^ and use this information to train and test the model defined below in Section 3.

## 3 METHODOLOGY

### 3.1 Overall model workflow

**FIGURE 2:**
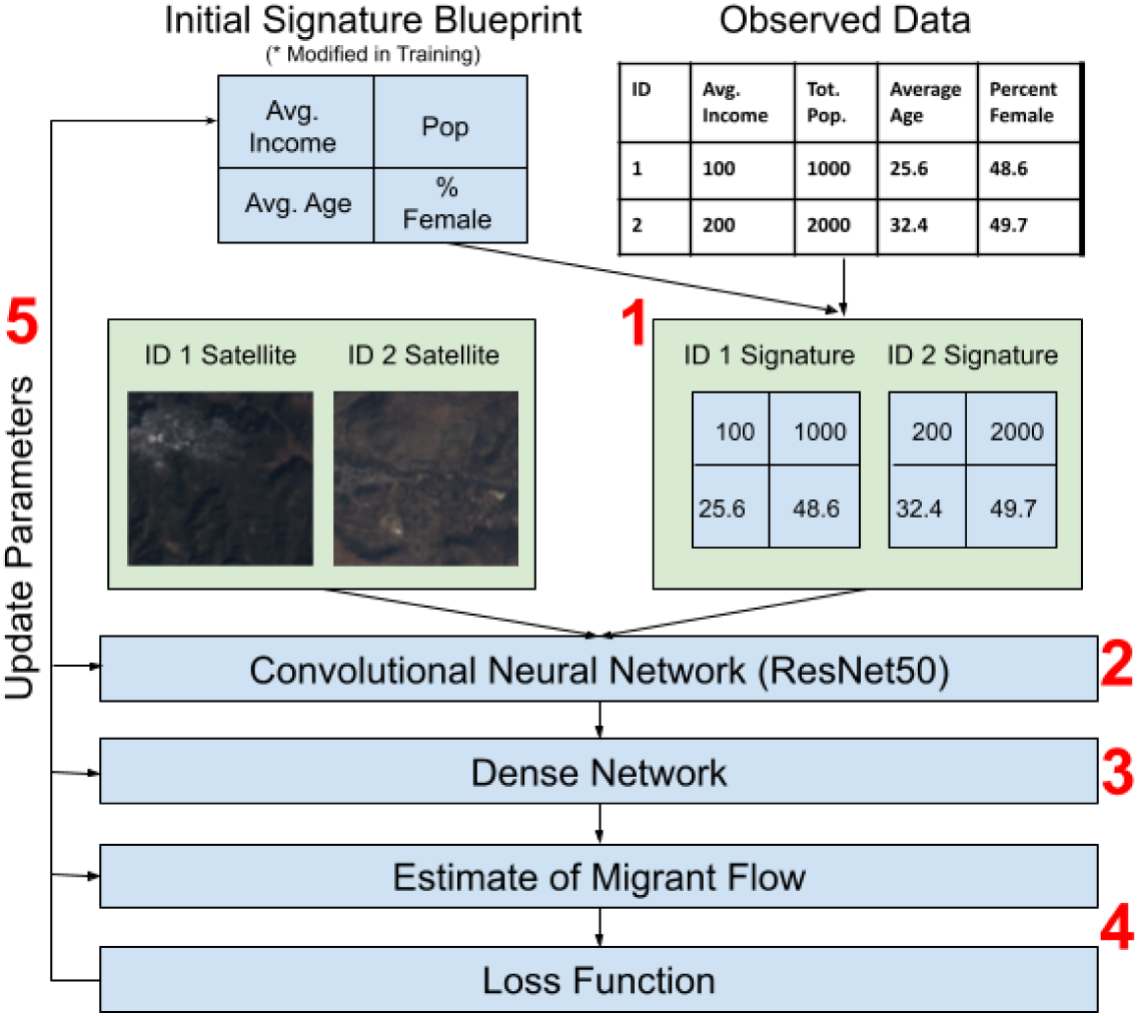
Overall modeling approach. In addition to parameters in the dense and convolutional network, the signature blueprint is updated on the basis of the loss function results, allowing for a flexible re‐arrangement of input observation data into an optimal 2D representation of the tabular data.

**FIGURE 3:**
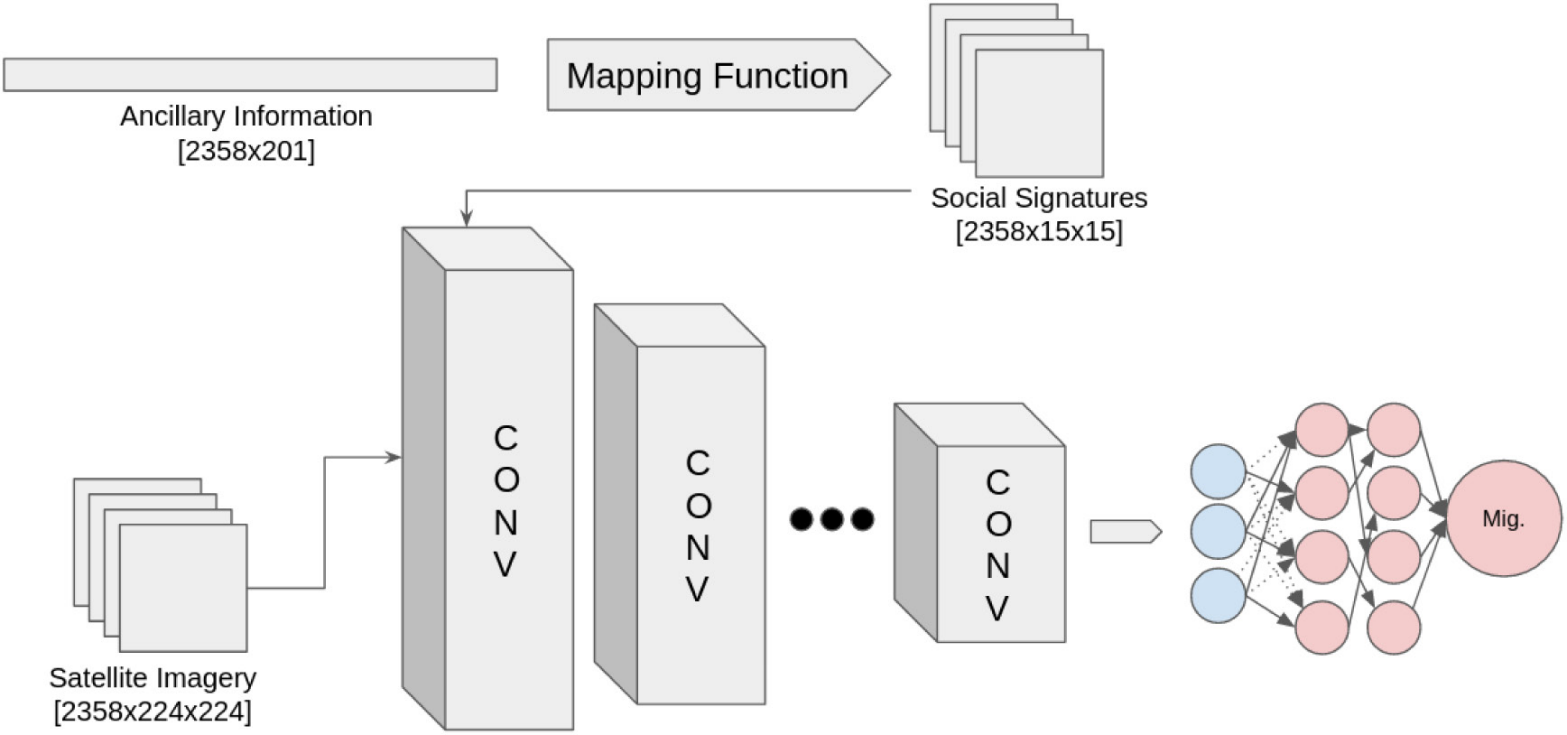
Flow of data through model architecture. Of note, the mapping function is parameterized, allowing the social signatures to be updated across epochs.

Figure 2 provides an example of the overall model workflow presented in this work, and Figure 3 provides an overview of data inputs and outputs into various model components. The approach we leverage follows a series of distinct steps, with the overall goal of: (a) constructing a social signature using the input census data by finding the optimal mapping of 1D tabular data to a 2D space; and (b) using this information in our estimation by feeding the resultant 2D image into a convolutional model. The specific steps are as follows:

1. Apply a transformation to our tabular (1D) municipality data, moving it into 2D space according to a parameterized mapping (we refer to the output 2D matrix as a “social signature”).
2. Apply a convolutional neural network [ResNet18 (He et al., 2016)] to the input satellite data and the generated social signature. In this step, the social signature is effectively treated as if it was any other image, and filters are convolved across it.
3. Pass the output vectors into a dense network.
4. Calculate an estimate of migrant flow from each municipality, and related losses.
5. Backpropagate throughout the network to update weights, including parameters which control the two‐dimensional positioning of each column of our observed tabular data in the social signature.
6. Repeat this procedure until parameter optimization is obtained.

By backpropagating to the social signature surface, we allow the network to construct an optimal image representation of the underlying tabular data. We discuss this unique aspect of the approach further in the next section.

### 3.2 Optimizing the social signature

**FIGURE 4:**
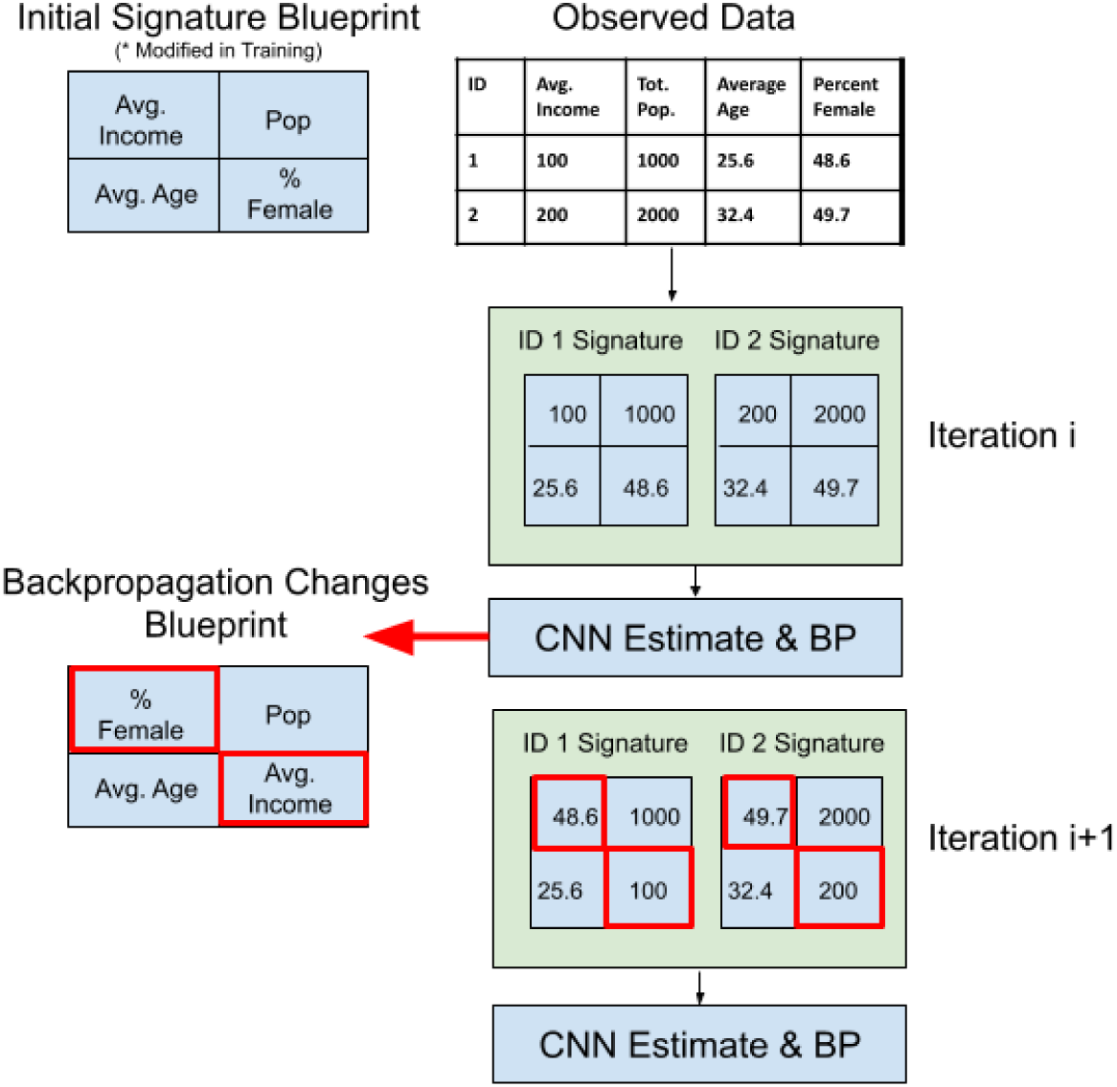
Example of the optimization procedure for the social signature.

A core contribution of the presented work is taking—for each unit of observation—the vector of observed socioeconomic variables and remapping them into a 2D space (see Figure 4). The idea of mapping 1D descriptors of an object to 2D space arose in genomics literature (Sharma et al., 2019), in which the structure of a gene provides a natural mapping. Despite facing a similar challenge (i.e., hundreds of covariates that are inter‐related with one another), in our application, we have no such mapping—that is, it is not clear if data on (for example) average income should be placed in close proximity to population, or if another structure might be more appropriate. Without identifying an optimal “blueprint” with which to map our socioeconomic data to two‐dimensional space, we run the risk of losing many of the benefits of this mapping (in particular, the capability to mitigate sparse or heavily correlated data).

Recognizing the importance of this mapping, we integrate the mapping itself as a parameter in our network, as summarized in Figure 4. At the initial state of the network, we first define a random mapping, that is, in the case of four ancillary variables, we would randomly allocate one of these four to a single cell of the 2D matrix (initialized with the smallest square dimensions possible to contain all variables). This procedure scales, that is, in the case of 201 variables, a 2D representation of 201 cells would be constructed with a random initialization, with the goal of mapping these cells to an optimal organization during the optimization procedure. This mapping is then fed forward through the convolutional network, and the ordering of variables is updated based on the accuracy (or lack thereof) of the final estimate. We ultimately seek to identify the single mapping that minimizes the overall loss of the CNN.

This approach is formalized as follows. We define *X* as a vector of ancillary data with length *A* (i.e., the number of dimensions in the ancillary data), in which each element *X*_*i*_ is to be mapped to a single cell within matrix **S** of size $\left\lceil \sqrt{\left(A\right)}\right\rceil x\left\lceil \sqrt{\left(A\right)}\right\rceil$. **S** represents the social signature we seek to construct to input it into the convolutional stages of the network. Additionally, we define an indexing vector, *B*, which is used to define a blueprint that maps the one‐dimensional vector *X* to *S*. Vector *B* has an identical length to *X*, and is initialized with random values *B*_*i*_. Finally, vector *T* is a holding vector with identical length to *X*.

During the first forward pass of the network, matrix **S** is constructed through a multiple‐step procedure, in which:

1. Vector *X* is sorted into *T* in ascending rank order on the basis of the values in vector *B*. For example, in the case of *i* = 10, if *B*_10_ is the largest value in vector *B*, *X*_10_ is mapped to *T*_1_.
2. Vector *T* is reshaped to a shape of $\left\lceil \sqrt{\left(A\right)}\right\rceil x\left\lceil \sqrt{\left(A\right)}\right\rceil$, in which each element *T*_*i*_ is entered into the matrix starting with the upper‐left value, and winding left‐to‐right.
3. **S** is set equal to the reshaped *T*.

The resultant matrix **S** is then fed forward into the convolutional stages of the network, and the values in vector *B* are added to the list of parameters to be updated during backpropagation to facilitate the identification of an optimal mapping. An upside of this approach is that, during backpropagation, only the values in vector *B* need to be updated—represented as blueprint changes in Figure 4. Because only one element is added to *B* for each input ancillary dataset *X*_*i*_, the overall number of additional parameters that are required to be fit in the network is limited to *A*, although alternative network architectures may necessitate values larger than *A*.

### 3.3 Implementation and validation

To illustrate the value of integrating information using a social signature, we perform four separate tests and present the accuracy of each in our results. The specific tests we perform are as follows:

- *Dense Net*. A four‐layer neural network in which each of the socioeconomic variables are input into the network and a single output (migration) is predicted.
- *Satellite Imagery Model*. A ResNet50 (pretrained with ImageNet) convolutional neural network using 12 months of satellite imagery from 2010 as input. No socioeconomic variables are used in this baseline model.
- *Social Signature without Imagery*. The social signature model detailed in Section 3, omitting satellite imagery.
- *Social Signature with Imagery*. The full model described in Section 3, incorporating the social signature and satellite imagery.

Tests were implemented using 8 NVIDIA RTX6000 GPUs and pyTorch version 1.8.1. For each test, the data being trained on is the *N* = 2358 municipalities in Mexico, using a 80/20 train/test split; *z*‐score standardization is applied to all input information. We present both the *R*‐squared (*r* ^2^) and mean absolute error (MAE) for each of these cases; MAE is used as the minimization target for optimization. Each model using ancillary data includes the variables presented in the Appendix 1. Hyperparameters were tuned independently in each case, and additional epochs performed until no further improvements in loss could be achieved (generally achieved between 200 and 250 epochs for the presented learning rates and problem scope). Learning rates, batch size, and Adam optimizer beta parameters were all selected through a series of systematic tests in which each hyperparameter was modified independently until an optimal performing value was found (using the imagery‐only model as the baseline). Learning rates and batch sizes for each test are shown in Table 2; an Adam optimizer with betas of 0.5 and 0.9 was selected.^3^

**TABLE 2:** Summary of accuracy of estimates for each modeling strategy

Model	Test *r* ^2^	MAE	Number of epochs	Learning rate	Batch size
Social signature with imagery	0.72	913	250	0.001	64
*Comparison models*					
1. Dense Net	0.627	1019	250	0.001	64
2. Social Signature without Imagery	0.662	959	250	0.001	64
3. Satellite Imagery Model	0.467	4547	200	0.01	64

## 4 RESULTS

The results of all model tests are summarized in Table 2. Of the 2358 municipalities included in the analysis, estimates were generated for a total of 1944 after removing municipalities with insufficient imagery due to cloud cover. These municipalities were largely localized to two regions, including a portion of suburbs of Mexico City and rural regions around Chiapas.

The first tested model was a fully connected network with four layers. The input shape of 201 was passed forward into hidden layers with sizes of 128, 64, and 32, respectively. No activation functions were integrated, providing a baseline accuracy that might be expected using socioeconomic information alone. After 250 epochs of training, this model achieved a *r* ^2^ of 0.63, and a mean absolute error (MAE) of 1019.

The second tested model built on the dense net approach, first applying the social signature construction routine detailed in Section 3.2 to the socioeconomic data, and then passing the constructed signatures into a ResNet18. This reprojection of the data from the 1D vector of covariates to the 2D signature resulted in a small improvement in *r* ^2^, increasing to 0.66. The MAE also decreased to 959.

The third comparison model included only satellite imagery, using a ResNet18 and the imagery from a given census unit (i.e., determining how well satellite imagery alone could predict migratory trends). As expected, this was the worst performance of the test cases, with a *r* ^2^ of 0.47 and a MAE of 4547.

**FIGURE 5:**
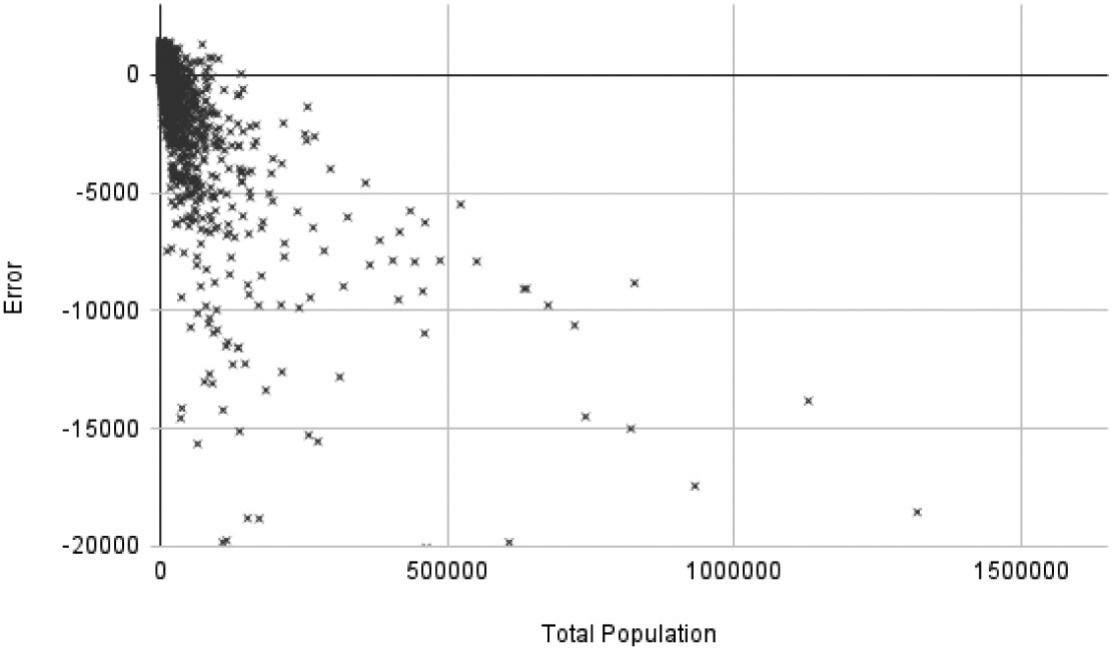
Scatterplot contrasting the overall error ($\hat{y}-y$) of the estimate of international migratory flows for each municipality to the municipalities population (*r* ^2^ = 0.66). Outliers omitted from visualization, but included in calculation of *r* ^2^. The model tends to under‐estimate flows from municipalities with large populations.

The full social signature with imagery outperformed all baseline cases, with a *r* ^2^ of 0.72 and MAE of 913. Approximately 64% of estimates were accurate to within 1000 migrants for a given flow; 38% were accurate to within 500 migrants. Additionally, as a secondary analysis, we explored how error correlated along the various dimensions available in our dataset (Table 2). As was expected, we observed no spatial pattern in our errors. However, total population was the most closely correlated with error, with a *r* ^2^ of 0.66 (see Figure 5). We discuss some of our model optimization strategies in the next section, and how these strategies are inter‐related with this apparent bias in model estimates.

In addition to these results, we performed additional tests to explore the degree to which errors may be correlated across space (thus indicating a lack of accounting for either spatial dependence or ancillary information with spatial correlations). A Moran's *I* was estimated on the basis of the surface of errors, using a first‐order Queen's contiguity matrix. Results suggested little evidence of spatial correlation in errors, with a global Moran's *I* value of 0.153. A local Moran's *I* indicated some evidence (*p* = 0.05) of significant error clustering in and around the Chiapas & Tabasco region, to the southwest of the Yucatan peninsula.

## 5 DISCUSSION

While the social signature model showed the highest performance of all tested cases, there are still marked limitations of the presented approach. As Figure 1 shows, the predicted pattern of where migrants are originating from is broadly similar to the observed data, but with a number of notable exceptions. While the model is capable of predicting migratory flows are more likely from the areas in and around Mexico City, it is unable to capture the extremes; similarly, it rarely identifies cases of extremely low migration in rural areas. Figure 5 further explores the relationship between error and total population, indicating that the currently specified model tends to under‐estimate in areas with higher population (i.e., the same areas in and around Mexico City). Because total population is included in the model, this suggests that a larger sample size and/or deeper network architecture would likely be beneficial to allow the model more observations with which to identify optimal parameters.

To better understand the mechanisms driving the presented model, we further apply a measurement of feature importance—specifically, permutation feature importance [sometimes referred to as model reliance (Breiman, 2001, Fisher et al., 2018)]—to explore the relative importance of different covariates in the presented model. The fundamental concept of permutation feature importance is that if a data dimension is unimportant to the model, randomly shuffling the values of that dimension would have little impact on overall error (and, conversely, shuffling the data of important dimensions would increase error). Explicit details of how permutation feature importance is implemented with convolutional models can be found in Fisher et al. (2018).

In our implementation, we iteratively loop over each of our 201 variables, in each case permuting the data in that variable and running the fully fit social signature model on this revised input data. We then record the overall change in mean absolute error in each case, and define feature importance as a quotient (Fisher et al., 2018):

(1) $${\mathrm{FI}}_{j}={\mathrm{MAE}}_{\text{permuted}}/{\mathrm{MAE}}_{\text{original}}$$

where each dimension of the ancillary data *j* is assigned a feature importance quotient (FI) by dividing the mean absolute error of the estimate after permutation is done by the original.

**FIGURE 6:**
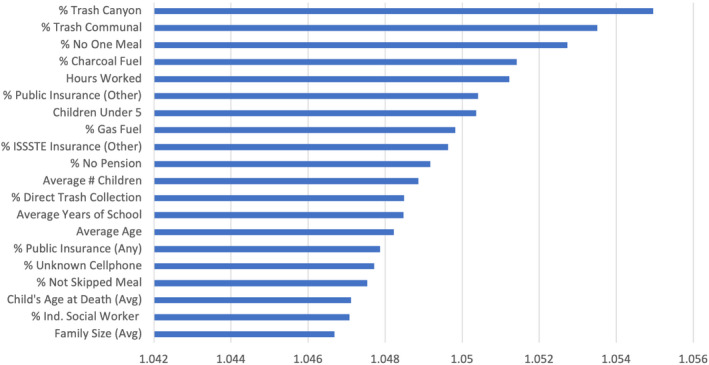
Top 10 permutation feature importance values in social signature deep learning model.

The results from the PIF are presented in Figure 6. The data suggest that basic municipal infrastructure parameters (trash collection and the type of fuel used for cooking), health (health insurance, food), economic conditions (i.e., hours worked, education level), and demographic characteristics (i.e., age and family structure) have the strongest effects on the migratory outcomes predicted. These findings are consistent with the well‐established literature on prevalent models in migration theory as they were referenced in Section 1 of this article. For example, correlations between increasing age and migration have been identified in past literature on migratory flows from Mexico (Nawrotzki et al., 2013), likely reflective of a similar reduction in capacity to migrate as age increases.

Moving beyond the importance of individual variables, interpretation of the optimal social signature identified can help provide information on how information is interrelated as it relates to migration. As the location of each ancillary variable is parameterized in the surface itself (see Figure 4), the final pattern of the derived signature can help inform us as to groupings of variables that may have important interrelationships or correlations within them. In the implementation presented in this article, we used a 3 × 3 filter to convolve across the generated signature, so groupings of variables within 3 × 3 regions are of particular interest. While it is not possible to identify all relationships across these groupings that may occur at deeper levels of the network, visualizing the surface can provide top‐level information about potentially meaningful clusters.

**FIGURE 7:**
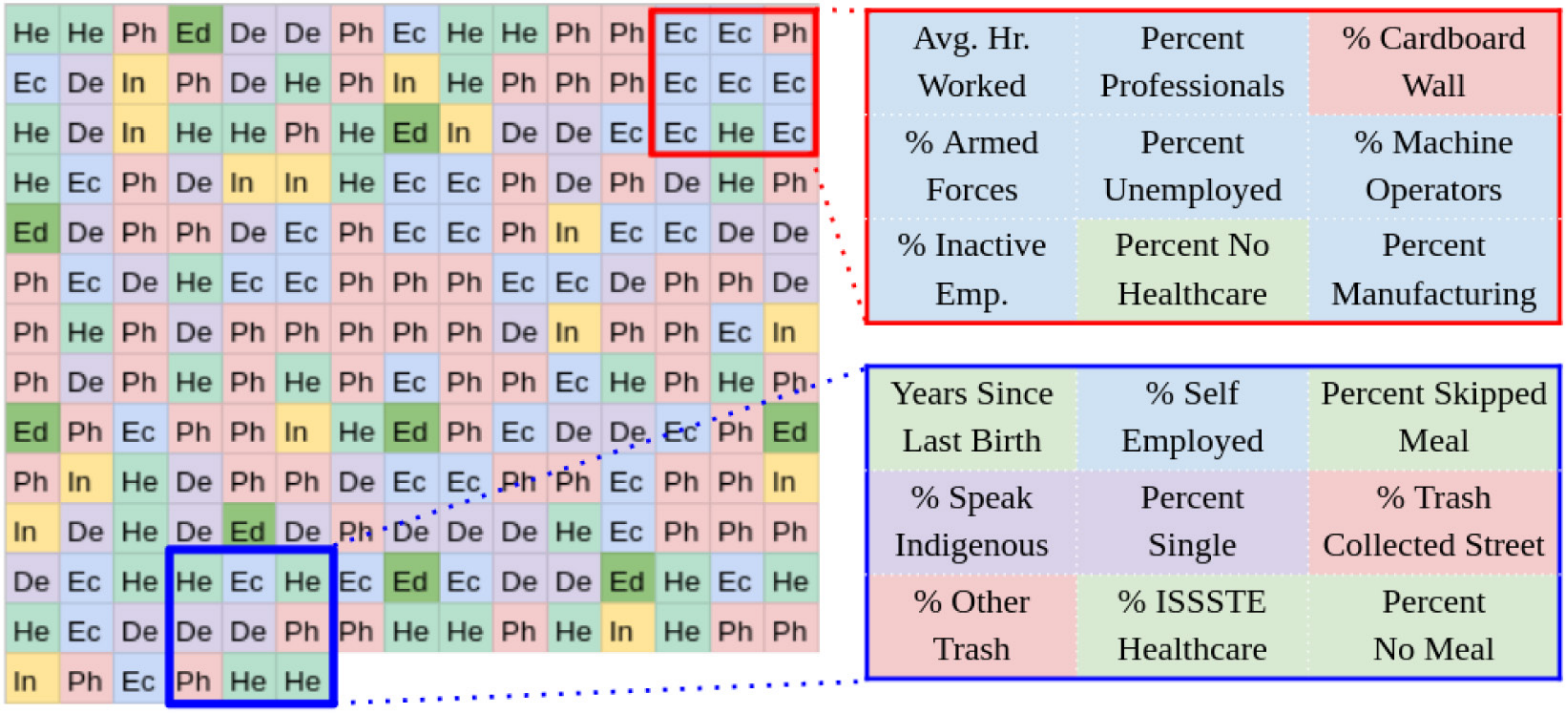
Final social signature surface generated. Each cell represents one of the 201 variables included in the analysis, colored according to type (light green: Health; red: Physical house; dark green: Education; purple: Demographics; yellow: Information; blue: Economic.

**FIGURE 8:**
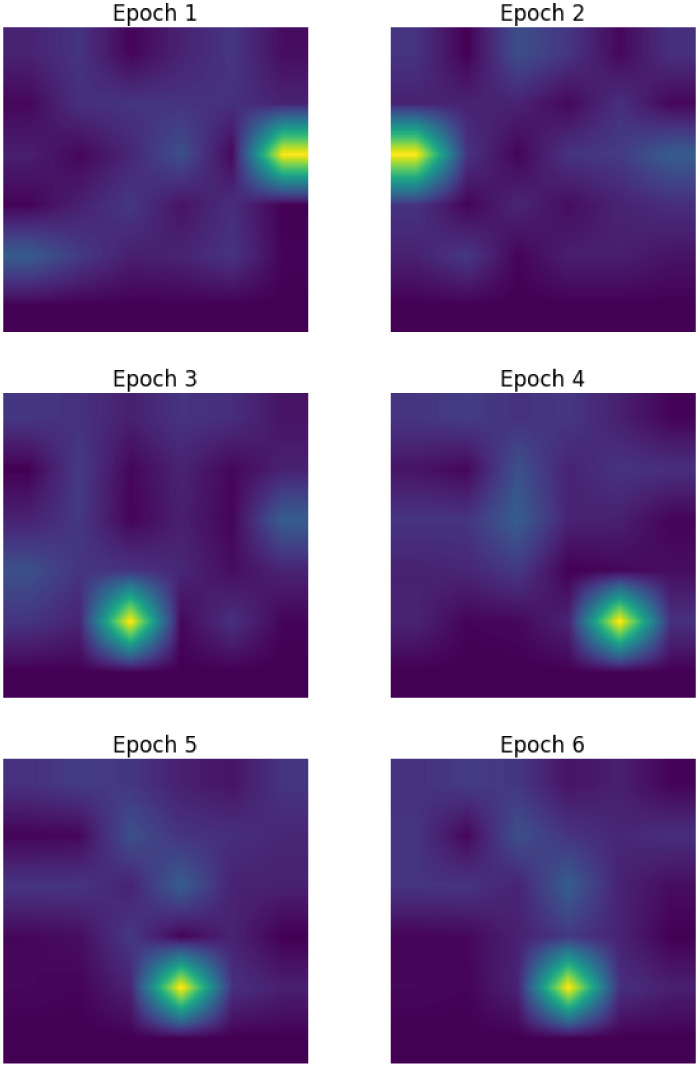
Example of how the social signature shifts across epochs within the network. The highlighted bright element is the representative of how the “Total population” variable was remapped across epochs.

Incorporating the social signature mapping as a parameter within the network resulted in substantial changes in the arrangement of the social signature itself throughout the model. Because the social signature is ultimately represented as an image, we can observe the ways in which the pixel values fluctuate from epoch to epoch within the network (ultimately resulting in the final image layout seen in Figure 7). Figure 8 shows one example of the evolution of a social signature across model epochs, with each figure showing the values of the social signature at the end of a given epoch.

Figure 7 shows the signature derived in the final model presented in this work. Two regions of the signature are highlighted as exemplars of the approach, and the type of information that can be gleaned. First, the red box in the upper right illustrates the grouping of variables inter‐related with unemployment, a lack of healthcare, and total hours worked. The dynamic grouping of these variables through the parameterization strategy shown in Figure 4 suggests that the co‐occurrence of certain values along these nine dimensions is of importance in generating an accurate prediction. Here, we can suggest that the *interrelationship* between unemployed populations and the percentage of individuals with no healthcare plays a meaningful role in driving migratory flows. Similarly, the blue box highlights a region which contains information on healthcare, meals skipped, and trash collection, indicating a separate set of possible inter‐dependencies. These examples serve to highlight the potential of social signatures for understanding drivers, but are of limited value due to the limitations inherent to the predictive models presented here. Considerable future research could explore this signature interpretation approach further in causal attribution contexts—incorporating metrics of model influence, for example, or ascertaining significance by removing individual elements of the data frame and re‐running the model, recording reductions in accuracy.

Researchers seeking to leverage approaches similar to the signature described here should be aware of limitations in our current understanding of the field, many of which provide fertile ground for future inquiry. First, the mapping technique described in this piece relies on a single function, meaning that socioeconomic information is always mapped onto a square “image” with dimensions $\sqrt{\left(A\right)}\;x\;\sqrt{\left(A\right)}$ (where *A* is the number of attributes). No research exists today on what the most appropriate mappings for this might be, that is, different network filter dimensions or mappings may be most appropriate for this type of data. Similarly, the winding order strategy most appropriate for 2D mapping is unclear, and the implementation in this article will result in a nonlinear relationship in edge cases (i.e., when a value previously located in the 15th column of a 15 × 15 image is moved to the 1st column, the Euclidean distance of the movement is larger than if it shifts from the 14th column to 15th column). Second, there are unique challenges associated with model explanability in the context of this work; while methods such as permutation feature importance can provide insight into the relative impact of different attributes, traditional techniques to visualize the impact of features within convolutional network architectures do not consider tradeoffs between mapping weights and filter weights, leaving a potential avenue for future research.

## 6 CONCLUSION

In this article, we presented a deep‐learning based data fusion technique to estimate migratory flows from Mexico to the United States. We find that migratory flows can be estimated at the municipality scale with an accuracy of *r* ^2^ = 0.72, improving on models which leverage only socioeconomic information by approximately 10% (an improvement in *r* ^2^ of 0.1).

Our findings make three main contributions to the literature. First, we present a novel approach to integrating socioeconomic and satellite data to improve our capability to predict migratory flows, illustrating the capacity of a social signature approach to improve predictive capabilities. Second, we provide further evidence of the value of satellite imagery and convolutional neural networks for estimating migratory flows, expanding on literature using satellite imagery to predict socioeconomic variables more broadly. Third, we provide some evidence that many of the drivers of migratory flows identified in the broader literature can also be identified as key drivers in deep learning models.

## FUNDING INFORMATION

U.S. Department of Homeland Security, Center for Accelerating Operating Efficiency, Arizona State University. Grant Award Number 17STQAC00001‐03‐03.

## CONFLICT OF INTEREST

The authors declare no conflict of interest.

## Data Availability

The data that support the findings of this study are available from IPUMS International at https://international.ipums.org/international/.

## Endnotes

^1^
While no specific information on whether an international migrant was US‐bound exists in the Mexican census, as of 2019 approximately 97.4% of all Mexican emigrants' destination was the United States (Ng et al., 2020). While this strategy omits single‐family households in which all members of the household moved, the population we are most prone to undercount (single‐person households) represent only 3% of households in Mexico; 32% of families live with extended family.

^2^
We create 224 × 224 tiles so as to be able to optimally take advantage of previous weights trained on ImageNet in our transfer learning stage, and avoid any data loss due to image warping. Each image is weighted in the final model according to the total number of images for a municipality so that all images carry an equal weight, and the final estimate is calculated by averaging all inputs.

^3^
All details on our implementation strategy can be seen in our replication code made available online at https://github.com/DanRunfola/socialSignature_MX_Migration. Please note that we are unable to redistribute the source data census information for this analysis due to license agreements; however, interested users can retrieve this information from ipums.org at no monetary cost.

## APPENDIX 1 COMPLETE LISTING OF ALL VARIABLES USED IN ANALYSIS

This Appendix provides a full list of all variables used in this analysis, as well as the groupings leveraged in producing figures throughout the document. Also provided are the parameterized mapping values for the construction of the social signature (and concomitant ranking for the mapping function).

**Table:**

Variable group	Variable name	Social signature parameterization	Social signature blueprint mapping location
Demographic	total_pop	60.9823	58
Demographic	perc_rural	9.1412	100
Demographic	unrel_ppl	49.9742	65
Demographic	perc_one_person_hhtype	210.0583	20
Demographic	perc_married_no_children_hhtype	214.7408	17
Demographic	perc_married_with_children_hhtype	16.5928	87
Demographic	perc_single_parent_hhtype	55.009	62
Demographic	perc_extended_family_hhtype	93.6725	41
Demographic	perc_composite_hhytpe	28.1091	78
Demographic	perc_non_family_hhtype	−43.3635	131
Demographic	perc_unclassifiable_hhtype	−62.6992	139
Demographic	avg_nfams	−99.6938	159
Demographic	avg_ncouples	83.2246	49
Demographic	avg_nmothers	−92.0229	156
Demographic	avg_nfathers	451.6351	5
Demographic	avg_npersons	−89.2188	152
Demographic	avg_eldch_age	−167.3317	176
Demographic	avg_yngch_age	−162.8154	175
Demographic	perc_single	−244.5899	185
Demographic	perc_married	−114.1688	166
Demographic	perc_separated	−214.5356	183
Demographic	perc_widowed	65.3096	56
Demographic	perc_marriage_unknown	37.7299	75
Demographic	perc_native_born_nativity	−102.2147	160
Demographic	perc_foreign_born_nativity	1.3754	107
Demographic	perc_yes_indig	−66.916	142
Demographic	perc_no_indig	120.6098	32
Demographic	perc_yes_speakind	−224.6967	184
Demographic	perc_yes_and_spanish_speakind	97.2841	40
Demographic	perc_yes_only_indig_speakind	−45.4091	132
Demographic	perc_no_speakind	−90.9219	154
Demographic	avg_age	−97.2522	158
Demographic	perc_urban	40.5623	74
Demographic	avg_famsize	391.0973	6
Demographic	avg_nchild	13.1593	94
Demographic	avg_nchlt5	15.2249	90
Economic	sum_income	−12.0844	116
Economic	sum_earned_income	−23.5427	123
Economic	weighted_avg_income	40.8963	73
Economic	weighted_avg_earned_income	−73.1683	144
Economic	perc_owned	−103.131	162
Economic	perc_not_owned	42.8174	69
Economic	perc_employed	−383.4019	198
Economic	perc_unemployed	134.5314	29
Economic	perc_inactive_empstat	87.1225	43
Economic	perc_unknown_empstat	−178.6864	179
Economic	perc_senior_officials	−43.1239	130
Economic	perc_professionals	256.3073	14
Economic	perc_technicians_associate_professionals	−48.5612	133
Economic	perc_clerks	17.1383	86
Economic	perc_service_workers	69.2939	53
Economic	perc_agri_fish_workers	−147.2469	172
Economic	perc_trades_workers	68.9685	54
Economic	perc_machine_operators	132.9778	30
Economic	perc_elementary_occupations	303.3344	8
Economic	perc_armed_forces	144.6595	28
Economic	perc_agriculture_fishing_forestry_indgen	−69.4024	143
Economic	perc_mining_extraction_indgen	26.3821	81
Economic	perc_manufacturing_indgen	85.6989	45
Economic	perc_electricity_gas_water_wm_indgen	224.4006	16
Economic	perc_construction_indgen	−211.5922	182
Economic	perc_wholesale_retail_indgen	−119.2095	167
Economic	perc_hotels_restaurants_indgen	49.1013	66
Economic	perc_transportation_storage_indgen	30.0519	77
Economic	perc_financial_insurance_indgen	−8.4872	113
Economic	perc_public_administration_defense_indgen	26.6483	80
Economic	perc_business_real_estate_indgen	−76.7899	147
Economic	perc_education_indgen	4.4532	104
Economic	perc_health_social_work_indgen	−158.0378	174
Economic	perc_private_household_services_indgen	17.3778	85
Economic	perc_self_employed	−140.0303	170
Economic	perc_wage_worker	88.9591	42
Economic	perc_unpaid_worker	84.3546	47
Economic	avg_hrsactual1	258.9368	13
Economic	perc_no_pension	43.9773	68
Economic	perc_no_disemp	41.6492	72
Education	perc_yes_school	−152.3744	173
Education	perc_no_school	−169.3654	177
Education	perc_no_literacy	−38.1843	128
Education	perc_yes_literacy	−91.7548	155
Education	perc_less_than_primary_edu	−17.6488	121
Education	perc_primary_edu	98.7114	38
Education	perc_secondary_edu	−50.9989	135
Education	perc_university_edu	464.379	4
Education	avg_YRSCHOOL	55.074	61
Health	avg_chborn	75.2778	52
Health	avg_chsurv	84.5255	46
Health	avg_num_years_from_last_birth	−135.9623	169
Health	avg_chdead	−13.6545	117
Health	perc_no_lab	296.8198	9
Health	perc_yes_lab	127.5875	31
Health	perc_disabled	59.7646	59
Health	perc_not_disabled	294.8391	10
Health	perc_social_security_imss_hlthfac	−3.9819	111
Health	perc_permex_defense_navy_hlthfac	−284.9268	191
Health	perc_public_workers_issste_hlthfac	511.6509	1
Health	perc_ministry_of_public_health_hlthfac	−132.7794	168
Health	perc_private_facility_hlthfac	−194.3963	181
Health	perc_other_hlthfac	−15.4401	119
Health	perc_no_facility_used_hlthfac	−3.1183	109
Health	perc_imss_only_hlthcov	−53.3301	138
Health	perc_issste_only_hlthcov	−102.9388	161
Health	perc_pemex_military_naval_hlthcov	−296.9604	193
Health	perc_public_insurance_hlthcov	−262.8056	189
Health	perc_other_hlthcov	−181.2531	180
Health	perc_imss_issste_hlthcov	−590.0114	200
Health	perc_imss_pemex_military_naval_hlthcov	13.9231	92
Health	perc_imss_public_insurance_hlthcov	100.3457	37
Health	perc_imss_other_hlthcov	205.7886	21
Health	perc_issste_pemex_military_naval_hlthcov	484.1331	2
Health	perc_issste_public_insurance_hlthcov	110.0921	34
Health	perc_issste_other_hlthcov	−89.5548	153
Health	perc_pemex_military_naval_public_insurance_hlthcov	−177.2138	178
Health	perc_pemex_military_naval_other_hlthcov	108.1588	35
Health	perc_public_insurance_other_hlthcov	172.4246	24
Health	perc_no_coverage_hlthcov	86.7607	44
Health	perc_no_onemeal	−144.5758	171
Health	perc_no_nomeal	−657.453	201
Health	perc_no_nofood	−261.7165	188
Health	perc_dead_lastbmort	−30.7434	127
Health	avg_agedeadyr	27.2295	79
Information	perc_no_phone	−51.8278	137
Information	perc_yes_phone	76.1931	51
Information	perc_yes_cell	−30.7331	126
Information	perc_no_cell	183.0066	23
Information	perc_no_internet	−291.6582	192
Information	perc_yes_internet	−89.2157	151
Information	perc_no_computer	79.024	50
Information	perc_yes_computer	98.2894	39
Information	perc_no_tv	3.3868	105
Information	perc_yes_tv	42.5552	71
Information	perc_no_radio	−328.3254	196
Information	perc_yes_radio	−85.6885	150
Information	perc_unknown_phone	214.6631	18
Information	perc_unknown_cell	110.2272	33
Information	perc_unknown_internet	8.8827	101
Physical	perc_yes_electricity	31.9531	76
Physical	perc_no_electricity	−17.8994	122
Physical	perc_no_piped_water	68.3394	55
Physical	perc_unknown_water_supply	62.6555	57
Physical	perc_sewage_system	−42.3599	129
Physical	perc_septic_tank	1.6494	106
Physical	perc_no_sewage_system	10.9967	97
Physical	perc_unknown_sewage	54.8446	63
Physical	perc_electricity_fuelcook	−94.7667	157
Physical	perc_other_fuelcook	14.8264	91
Physical	perc_trash_burned	−9.0464	115
Physical	perc_trash_buried	15.6413	88
Physical	perc_no_autos	10.167	98
Physical	perc_yes_autos	12.5058	96
Physical	perc_no_hotwater	−15.8516	120
Physical	perc_yes_hotwater	1.0462	108
Physical	perc_no_washer	−8.794	114
Physical	perc_yes_washer	−113.1417	165
Physical	perc_no_refrig	186.0179	22
Physical	perc_yes_refrig	20.1786	84
Physical	avg_room_num	45.9297	67
Physical	avg_bedroom_num	42.6516	70
Physical	perc_no_kitchen	−73.6021	145
Physical	perc_yes_kitchen	9.3369	99
Physical	perc_no_toilet	13.4695	93
Physical	perc_flush_toilet	6.3966	102
Physical	perc_non_flush_other_toilet	−104.5496	164
Physical	perc_no_bath	52.2919	64
Physical	perc_yes_bath	−51.561	136
Physical	perc_no_unfinished_floor	−3.8901	110
Physical	perc_cement_floor	163.3371	27
Physical	perc_other_finished_floor	103.5356	36
Physical	perc_scrap_wall	−77.133	148
Physical	perc_cardboard_wall	242.539	15
Physical	perc_wood_wall	−77.6535	149
Physical	perc_reed_bamboo_palm_wall	5.3882	103
Physical	perc_brick_stone_wall	83.5843	48
Physical	perc_adobe_wall	−66.7502	141
Physical	perc_mud_wall	−26.9473	125
Physical	perc_metal_asbestos_sheet_wall	−73.6166	146
Physical	perc_masonry_roof	−49.1074	134
Physical	perc_slate_roof	−6.1862	112
Physical	perc_asbestos_roof	329.8614	7
Physical	perc_sheet_metal_roof	−355.1543	197
Physical	perc_plant_materials_roof	22.3486	83
Physical	perc_wood_roof	−14.2654	118
Physical	perc_thatch_roof	12.7461	95
Physical	perc_scrap_material_roof	56.9434	60
Physical	perc_cardboard_roof	168.0385	25
Physical	perc_piped_inside_dwelling_watsup	−63.6969	140
Physical	perc_piped_shared_watsup	167.4444	26
Physical	perc_piped_within_building_watsup	15.6408	89
Physical	perc_public_piped_watsup	479.883	3
Physical	perc_gas_piped_utility_fuelcook	−256.4779	187
Physical	perc_gas_tanked_bottled_fuelcook	292.7046	11
Physical	perc_wood_fuelcook	−312.5053	194
Physical	perc_charcoal_fuelcook	267.6657	12
Physical	perc_unknown_fuelcook	25.6361	82
Physical	perc_trash_collected_directly	−25.0437	124
Physical	perc_trash_collected_indirectly	−104.1329	163
Physical	perc_trash_street	−250.9431	186
Physical	perc_trash_river	210.7923	19
Physical	perc_trash_canyon	−269.2986	190
Physical	perc_trash_communal	−321.3191	195
Physical	perc_trash_unknown	−452.2279	199
